# The chemical neighborhood of cells in a diffusion-limited system

**DOI:** 10.3389/fmicb.2023.1155726

**Published:** 2023-04-18

**Authors:** Juliana Gesztesi, Jared T. Broddrick, Timothy Lannin, Jessica A. Lee

**Affiliations:** ^1^NASA Ames Research Center, Universities Space Research Association, Moffett Field, CA, United States; ^2^College of Engineering, Northeastern University, Boston, MA, United States; ^3^NASA Ames Research Center, Space Biosciences Research Branch, Moffett Field, CA, United States

**Keywords:** microgravity, diffusion modeling, Michaelis-Menten, glucose uptake, *E. coli*

## Abstract

Microorganisms follow us everywhere, and they will be essential to sustaining long-term human space exploration through applications such as vitamin synthesis, biomining, and more. Establishing a sustainable presence in space therefore requires that we better understand how stress due to the altered physical conditions of spaceflight affects our companion organisms. In microgravity environments such as orbital space stations, microorganisms likely experience the change in gravity primarily through changes in fluid mixing processes. Without sedimentation and density-driven convection, diffusion becomes the primary process governing the movement of growth substrates and wastes for microbial cells in suspension culture. Non-motile cells might therefore develop a substrate-deficient “zone of depletion” and experience stress due to starvation and/or waste build-up. This would in turn impact the concentration-dependent uptake rate of growth substrates and could be the cause of the altered growth rates previously observed in microorganisms in spaceflight and in ground-simulated microgravity. To better understand the extent of these concentration differences and their potential influence on substrate uptake rates, we used both an analytical solution and finite difference method to visualize concentration fields around individual cells. We modeled diffusion, using Fick’s Second Law, and nutrient uptake, using Michaelis–Menten kinetics, and assessed how that distribution varies in systems with multiple cells and varied geometries. We determined the radius of the zone of depletion, within which cells had reduced the substrate concentration by 10%, to be 5.04 mm for an individual *Escherichia coli* cell in the conditions we simulated. However, we saw a synergistic effect with multiple cells near each other: multiple cells in close proximity decreased the surrounding concentration by almost 95% from the initial substrate concentration. Our calculations provide researchers an inside look at suspension culture behavior in the diffusion-limited environment of microgravity at the scale of individual cells.

## Introduction

The expansion of space travel by both space agencies and private companies, as well as current plans to send astronauts back to the Moon for the first time in decades, have recently increased the relevance and traction of space microbiology. Humans carry 10–100 trillion cells of microorganisms on and within our bodies (Ursell et al., 2012). As humanity travels to space, we have no say in whether or not microbes accompany us: they will (Lopez et al., 2019). Moreover, microbes in space are of interest for their potential role in sustaining human life during long-term missions. For example, microbes may play a role in the process of *In Situ* Resource Utilization (ISRU), as in biomining (Gumulya et al., 2022) or the conversion of methane waste into biomass (NASA Technology Transfer, n.d.). They will also likely be the first organisms employed in the *in situ* production of foods and pharmaceuticals, and in environmental life support systems (Berliner et al., 2021; Keller et al., 2021; Liu et al., 2021; McNulty et al., 2021).

Because microbes play a crucial role in our journey to space, numerous experiments have been carried out in spaceflight and in simulated microgravity experiments on Earth to characterize microbes’ biological response to space conditions. A particularly puzzling feature of the space environment is the effect of microgravity on microbes, as there is no evidence that bacteria can detect the force of gravity directly, but they have been shown to respond to altered gravity conditions (Horneck et al., 2010; Bijlani et al., 2021; Wang et al., 2022). Numerous studies have reported differences in gene expression, antibiotic resistance, and virulence in spaceflight (Nickerson et al., 2003; Wilson et al., 2007; Aunins et al., 2018). At the scale of the microbial cell, the primary effects of microgravity may be due to alterations in the physical conditions that surround them, such as differences in mass transport (Todd, 1989; Klaus et al., 1997, 2004).

**Figure 1:**
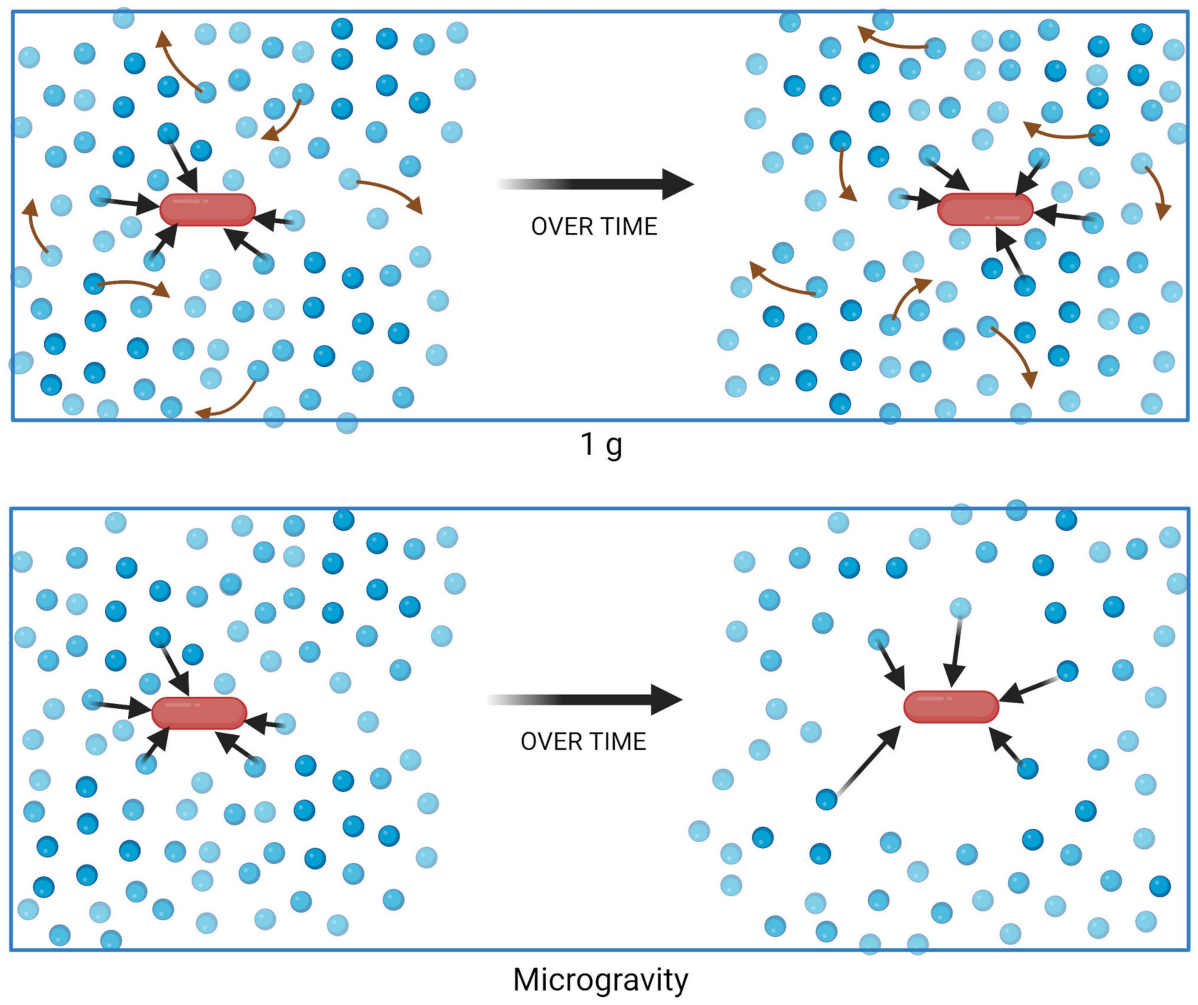
Nutrient uptake and distribution in a suspension culture in 1 g (top) and microgravity (bottom). Blue circles represent molecules of a growth substrate, consumed (black arrows) by the red bacterial cell. Red arrows represent mixing of substrate due to density-driven convection, which does not occur in quiescent fluids in microgravity. Figure generated using BioRender.

In liquid suspension culture, microbes experience a different physical environment in 1 g compared to microgravity. Within Earth’s gravity, mixing can occur as a result of two forces. First, natural convection occurs when density is not homogenous throughout the entirety of the fluid, due to factors such as temperature or solute concentration, and the force of gravity brings denser fluid downward. Second, passive diffusion leads to mixing based on concentration gradients. When density-driven convection brings low- and high-concentration regions of fluid in close contact, mixing may be rapid. However, in a quiescent fluid in microgravity, density-driven convection ceases to occur and the mixing of nutrients in the culture becomes diffusion-limited. Thus, it is theorized that a non-motile cell creates a “zone of depletion” surrounding itself as it consumes growth substrates, if nutrient uptake occurs faster than the substrate can diffuse into the zone (Figure 1) (Todd, 1989; Klaus et al., 1997, 2004). Analyses showing different responses from motile cells (which can disrupt the quiescent medium and/or escape the depletion zone) and non-motile cells support this theory (Benoit and Klaus, 2007). Similarly, the changes in mass transport in microgravity would also lead to waste product accumulation surrounding the cell, which could similarly contribute to the changes in phenotype discussed earlier.

Rotating Wall Vessels (RWVs), devices typically used to simulate microgravity for ground-based microbiology experiments, are designed to mimic the quiescent environment of microgravity by balancing forces to keep cells within their zone of depletion (Hammond and Hammond, 2001; Klaus, 2001). RWVs are a form of clinostat rotating in a 2-dimensional plane, and many forms have been used in experiments, including both deep cylindrical (Allen et al., 2022) and shallow wide (Chao and Das, 2015) devices. (Other microgravity simulation devices, such as 3D clinostats and random positioning machines, involve more complex fluid dynamics and are not discussed here (Wuest et al., 2017). And while there are many spaceflight settings in which cells are in dry, surface-attached, or mechanically mixed environments, the fluid environment is relevant to numerous applications, from fundamental biological experiments in CubeSat microfluidic culture devices (Bluck, 2007; Ricco et al., 2020), to bioreactors that omit stirring for ease of operation and reliability, such as those used for the BioNutrients project (Tabor, 2022), or those that employ perfusion technology to increase mass transport (Mozdzierz et al., 2015).

Unfortunately, it is difficult to test the depletion zone theory directly, since doing so would require measuring concentrations of nutrients directly surrounding an individual cell. Therefore, we explored computational modeling as a means to calculate the theoretical magnitude, extent, and temporal dynamics of this nutrient-deficient zone for single and multiple cells, and its potential effect on cells’ nutrient uptake rates. Similar work has previously been carried out in the context of understanding nutrient uptake by marine plankton; however, these works have typically focused on individual cells (Lazier and Mann, 1989; Karp-Boss et al., 1996). We recognized that space applications may involve growing microbes in suspension culture to densities at which cells will be within a few micrometers of each other (for example, at a typical experimental population of 10^7^ cells/mL, cells are on average only 46 μm apart), and therefore we sought to quantify how each cell’s nutrient consumption might affect its neighbor. Understanding chemical gradients can provide insight into their potential importance as one of the mechanisms behind microbial behavior in spaceflight and enable more accurate simulations of microgravity in ground-based experiments.

## Materials and methods

### Application of Fick’s second law

The first equation incorporated in this model is Fick’s Second Law, which describes the diffusion of a solute in a fluid medium. This is given in Equation (1), where *φ* is the concentration of the solute, *t* is time, and *D* is the diffusivity of the solute in the given medium. The partial derivative of *φ* with respect to *t* (left-hand side) is related to *D* and the Laplacian (second spatial derivative) of *φ* (right-hand side).


(1) $$\frac{\partial \varphi }{\partial t}=D\nabla^{2}\varphi .$$

### Analytical solution: “One cell in infinite medium”

First, we tested an analytical solution derived from Fick’s Second Law that describes diffusion from a continuous source in infinite media (Crank, 1979), shown in Equation (2). In our system, the source takes a negative value (therefore, acts a sink), and represents the uptake rate of nutrient by the cell. As in Equation (1), *φ* represents the concentration of solute at a given point (mmol); *D* is the diffusivity of a specific solute in a specific medium (cm^2^/s); and *t* is the time since the source’s start (sec). *V*_max_ denotes the value for this source (mmol/g/h, originally, but we converted to mmol/cell/s by multiplying by the average weight of an *Escherichia coli* cell and dividing by the number of seconds in an hour). *r* is the radial distance from the source (cm). We used parameters corresponding to non-motile *E. coli* cells growing in M9 glucose medium (see “Parameters” below) to calculate *φ* at each value of *r* from 0.018 to 2 cm in steps of 0.018 cm, for each value of *t* from 0 to 2 days in steps of 1 s.


(2) $$\varphi =\varphi +\left(\frac{V_{\max}}{4\pi *D*t}\right)*erfc\left(\frac{r}{2\sqrt{D*t}}\right).$$

### Finite difference method: “One or more cells per slice”

In order to be able to model multiple cells and to incorporate temporally dynamic feedback between uptake rates and concentrations, we used Finite Difference Method (FDM) to solve Fick’s Second Law at each time step. FDM allows for the approximation of partial differential equations by utilizing boundary and initial conditions. Space and time are discretized into finite steps, and the solution at each discrete step is approximated using algebraic equations containing finite differences and values from nearby points. FDM is applied in Equation (3), which is an expansion of Equation (1) in 2D Cartesian coordinates (*x*, *y*).


(3) $$\frac{\partial \varphi }{\partial t}=D\left(\frac{\partial^{2}\varphi }{\partial x^{2}}+\frac{\partial^{2}\varphi }{\partial y^{2}}\right).$$

The parameters *φ*, *t*, and *D* are as previously stated. The right hand of the equation is expanded to account for diffusion in each cartesian direction.

It should be noted that our FDM implementation models in pseudo-2D, where our system is essentially a repeating slice of 3-D space. Physically, we are showing a cross-section of a 3-D system with definite *x*, *y* bounds, but an indefinite *z* axis. We assume that the concentration gradient with respect to the *z* axis is zero: materials are equally likely to diffuse in either *z* direction. Therefore, we are solving for Fick’s Second Law in two dimensions, *x* and *y*, shown in Equation (3). This is equivalent to what we might expect if we had a string of cells, one per slice, where the slice repeats in both *z*-directions indefinitely.

In our model, the microbial cell was treated as a point source on the slice that takes in a finite number of moles per second, with the point of uptake located at the center of the cell. The algebraic equation used for FDM is given in Equation (4), representing the forward-time central-space (FTCS) explicit scheme.


(4) $$\begin{array}{l} \varphi_{\mathrm{new}}{}_{i,j}=D*\delta t*\\ \left(\frac{\left(\varphi_{i+1,j}-2*\varphi_{i,j}+\varphi_{i-1,j}\right)}{DX^{2}}+\frac{\left(\varphi_{i,j+1}-2*\varphi_{i,j}+\varphi_{i,j-1}\right)}{DY^{2}}\right)+\varphi_{i,j}. \end{array}$$

*Dx* and *Dy* are the size of the discretized space in the *x*, *y* directions, respectively. We carried out calculations using 180 μm as *δx* and *δy*. The size of the domain is 2 cm by 2 cm. *δt* is the time step and is set at 10 milliseconds. Values for *δx*, *δy*, and *δt* and the size of the domain were determined using convergence studies (Supplementary material). We modeled an *E. coli* cell and estimated it to be 1 μm in diameter (El-Hajj and Newman, 2015), so for visualization purposes we ignore the values for concentration within that space: for figures showing substrate concentrations, the value at the center of the cell (which was dictated by the concentration-dependent uptake rate) is displayed as an average of the four values surrounding it on the *x*, *y* plane, so that the plots do not show the discontinuity at the point of uptake.

### Application of Michaelis–Menten uptake dynamics

The second major component of the model describes a cell’s uptake rate of a nutrient. This rate is related to the surrounding concentration, as dictated by the classic Michaelis–Menten binding kinetics (Johnson and Goody, 2011) given in Equation (5). On a biological level, in the limit of large substrate (i.e., nutrient) concentration, all of the transport channels become occupied with substrate, so the limiting factor in uptake rate is the number of channels in the cell and the rate that they can transport, accounted for in the parameter *V*_max_ (in mmol/s). In the limit of small nutrient concentration, the uptake is limited by the receptor transport rate and the number of receptors, where these two factors are accounted for with parameters *V*_max_ and *K_m_* (in mM), and the concentration of substrate at the cell surface ( φ , in mM). Note that if substrate concentration, φ , is very high in comparison to the value of the Michaelis–Menten constant, *K_m_*, the rational expression in parentheses approaches 1, and the rate, *V*, approaches its maximum value, *V*_max_.


(5) $$V=V_{\max}\left(\frac{\varphi }{K_{m}+\varphi }\right).$$

The FDM model used in our study accounts for the interdependence of these two equations by alternately solving for the diffusion of nutrient and determining the Michaelis–Menten uptake rate per each time step. The analytical solution in Equation (2) does not account for the relationship in Equation (5), but rather assumes that *V* = *V*_max_ at all times.

### MATLAB computations

The process for the FDM model proceeds as follows: first, time and space are discretized, variables are initialized, and the system orientation (number of cells and layout) is chosen. Then, the model calculates the initial setup of the system, where the concentration at each *x*, *y* location is equal to the initial value. Next, the uptake rate for each cell is determined by the Michaelis–Menten equation, where substrate concentration is defined as the average of the four values surrounding the uptake point (cell center); that uptake rate is allocated to the cell center location. Then, the model uses FDM to calculate the concentrations at each *x*, *y* location at the next time step, after the cell consumes its pre-allocated amount based on the Michaelis–Menten kinetics at initial conditions. A new uptake rate is determined by the Michaelis–Menten equation using the new surrounding concentration average, and that rate is the new value for the cell center. The process repeats for each time step, until the model reaches steady state. Steady state is defined as the point at which the change in the average concentration at the cell surface is less than 0.1 μM per timestep, which was decided by the use of convergence studies (Supplementary material).

All computations were carried out and figures generated in MATLAB version R2022a (Update 2), with no additional add-ons or toolboxes. The code is provided in Supplementary material.

### Parameters

This model simulates a cell or multiple cells of non-motile *E. coli* growing in M9 minimal medium with 22 mM glucose (Cold Spring Harbor Protocols, 2010). This concentration was used for the initial values for the entirety of the space at the initial time step, and was the value fixed at the boundaries of the domain. Calculations were carried out in terms of absolute numbers of molecules rather than concentrations; the concentration 22 mM corresponds to 7.14 × 10^−9^ mmol per spatial segment as each has a discrete volume of 3.25 × 10^−10^ l. For ease of interpretation in reporting results, mmol were converted back again into mM by dividing by volume. Other parameters were as follows: *D*, the constant diffusivity of glucose in water, 600 μm^2^/s (Adam, 1946); *V*_max_, the maximum uptake rate of glucose by the *E. coli* in suspension, 10 mmol/g/h (Harcombe et al., 2014); and *K*_m_, the Michaelis–Menten constant of glucose uptake by *E. coli*, 1.75 μM (Natarajan and Srienc, 1999).

We were unable to find experimental data for the diffusivity of glucose in M9 minimal medium, so we used the diffusivity of glucose in water because the composition of M9 medium is greater than 99% (*w*/*v*) water (Cold Spring Harbor Protocols, 2010). The Stokes-Einstein equation describes the diffusivity of a particle in a solvent as a function of temperature (*T*), viscosity of the fluid (*η*), and particle radius (*r*), where *k*_B_ is the Boltzmann constant (Coglitore et al., 2017; Equation 6).


(6) $$D=\frac{k_{B}*T}{6\pi *\eta *r}.$$

This relationship applies when the system has a low Reynolds number, as in the quiescent environment of microgravity; and when the molecules of the solvent are much smaller than that of the solute, as for the water-glucose system [water measures about 2.8 Å (D’Arrigo, 1978), while the diameter of a ring-form glucose molecule is about 8.6 Å (Uri, n.d.)]. Given the Stokes-Einstein equation, the only factor that would result in a difference in *D* between M9 and water would be viscosity, η; and we assume this to be negligible, given the low concentration of solutes in M9. In addition, the molecular weight of M9 solutes is sufficiently small that we do not expect them to impact diffusivity substantially, since as solute size and complexity decrease, the impact on diffusivity decreases as well (Chan et al., 2015).

## Results

### One cell in infinite medium

**Figure 2:**
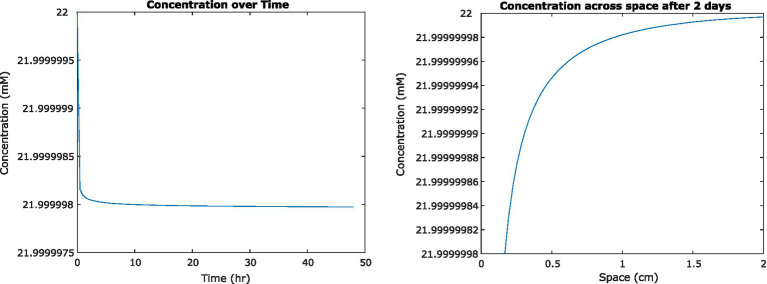
(Left) Concentration of glucose in medium at a distance of 0.2 mm from the center of a single *Escherichia coli* cell, as it changes over time. (Right) Concentration of glucose surrounding that cell at steady state, 2 days from initiation.

To examine the impact of nutrient uptake and diffusion on the fluid environment, we initially used a previously published analytical solution to model a single cell in suspension culture (Crank, 1979). We found that glucose concentrations surrounding the cell decreased by less than 100 nM. The definition of steady state we developed for the FDM model was therefore not applicable, but we found that concentrations appeared to converge on a steady state after approximately 2 days of simulation time (Figure 2). As mentioned previously, these results do not account for concentration-dependent cell uptake rates.

### One cell per slice

**Figure 3:**
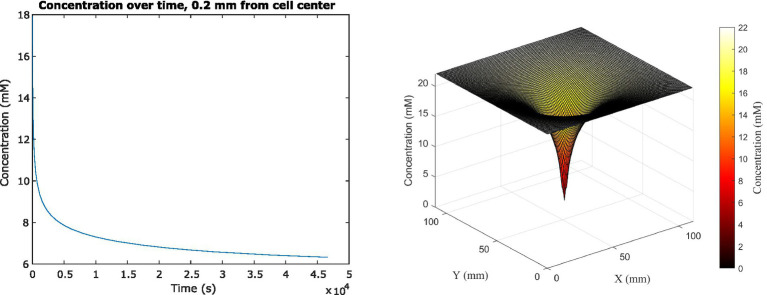
(Left) Average glucose concentration over time at a distance of 0.2 mm from the center of an *E. coli* cell in M9 medium. (Right) Surface plot showing glucose concentration across *x*, *y* space in the vicinity of the cell. Concentration is shown both on the *z*-axis and in the color gradient.

Because the analytical solution assumes a single cell in infinite medium with a constant glucose uptake rate, we had to use a different method to investigate scenarios more relevant to space biological applications. We therefore used the Finite Difference Method to track the change in glucose concentration surrounding the cell center dynamically, as it changed over time in feedback with changing cell uptake rates, until concentrations reached steady state (Figure 3). The system reached steady state at 46,440 s (or almost 13 h) of simulated time; at this time, the average concentration adjacent to the cell surface had decreased to 6.322 mM, which was a 71.3% decrease from the original 22 mM concentration in the medium. This surrounding concentration decreased the cell’s glucose uptake rate by 0.0277%. The depletion zone (which we defined as more than a 10% decrease in surrounding concentration) extended to a radius of 5.04 mm around the cell.

### Two cells per slice

**Figure 4:**
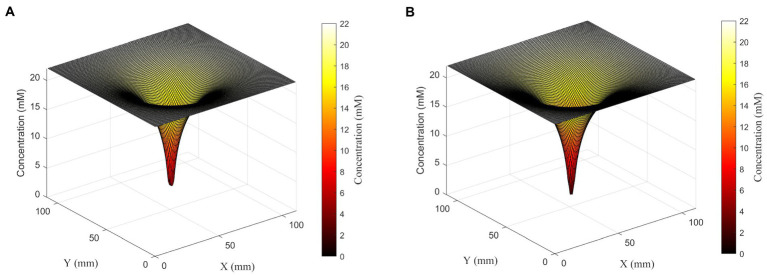
Surface plots showing glucose concentration field around a set of four cells in the linear configuration (left) or square configuration (right). Although cells are 360 μm apart from each other, cells in both configurations form one deep concentration gradient. Glucose concentration is shown both on the *z*-axis and in the color gradient.

We next examined how a cell can influence a neighbor’s environment by modeling pairs of cells at varying distances from each other, testing this model with cells located 2, 4, or 6 spatial steps apart (360, 720, and 1,080 μm apart). For all scenarios, we examined the change in uptake rate over time (following Michaelis–Menten kinetics), and at steady state we assessed the concentration surrounding the cell, the concentration between the two cells, and the size of the depletion zone around a cell (Table 1; Figure 4).

**Table 1:** Results from FDM calculation of glucose concentrations surrounding two non-motile *Escherichia coli* cells in M9 medium in diffusion-limited microgravity suspension culture, at varying distances from each other.

	1,080 μm apart	720 μm apart	360 μm apart
Average concentration 0.2 mm from cell center	4.024	3.537	4.408
Decrease from initial concentration	81.7%	83.9%	80.0%
Concentration at center between the cells (mM)	5.805	4.346	2.204
Size of depletion zone (radius, mm)	6.12	5.94	5.58
Steady state time point (s)	45,620	43,530	39,990
Decrease in uptake rate	0.0384%	0.0402%	0.0434%

We found that there is a synergistic effect in substrate uptake when two cells are close together: where an individual cell decreases its local glucose concentration to 6.322 mM before reaching steady state, two cells spaced 1,080 μm apart deplete it to 5.805 mM at the center between them (Table 1). And the effect of proximity increases with decreasing distance, up to the theoretical maximum of two adjacent cells (360 μm apart), which deplete their local glucose to 2.204 mM between them. However, the radius of each cell’s 10% depletion zone decreases when it gets nearer to its neighbors, to a minimum of 5.58 mm.

### Four cells per slice

**Figure 5:**
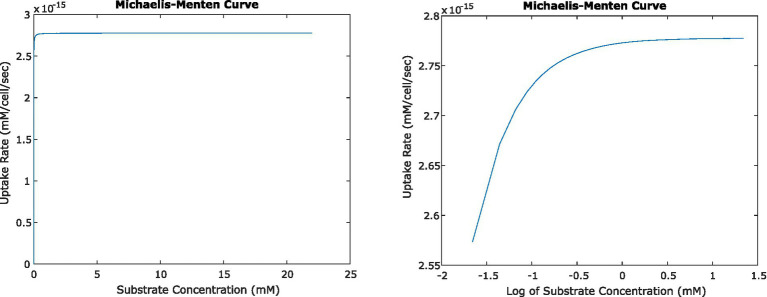
Relationship between uptake rate and substrate concentration for *E. coli* consuming glucose, based on Michaelis–Menten uptake dynamics, shown on a linear scale (left) and log-linear (right). At steady state, the activity of single cells and small numbers of cells result in surrounding concentrations that in turn result in uptake rates very close to *V*_max_.

To further explore the synergistic effects of multiple cells on local substrate concentrations, we performed a similar analysis on a four-cell system with different spatial distributions: four cells in a line with sequential separations of two spatial steps; and a square shape distribution where each cell is located at the square’s vertex, and the square has a side length of 2 spatial steps. We found that the square distribution led to lower glucose concentrations between cells (Table 2; Figure 5).

**Table 2:** Results from finite difference method (FDM) calculation of glucose concentrations surrounding four non-motile *E. coli* cells in M9 medium in diffusion-limited microgravity suspension culture, in different spatial configurations.

	Linear distribution	Square distribution
Average concentration at system center (mM)	1.606	1.194
Decrease from initial concentration	92.7%	94.6%
Size of depletion zone (radius, mm)	4.86	5.94
Steady-state time point (s)	21,190	31,220
Decrease in uptake rate	0.0549%	0.0715%

Note that “average concentration 1 μm from cell center” is an average of all the points at that radial distance, though concentrations may be lower on one side of the cell than the other.

### Glucose uptake rates

In all of the systems described above, the changing concentrations changed glucose uptake rates very little. Even at the closest orientation of two cells we tested, the uptake rate decreased by less than 0.1% from the original uptake rate at 22 mM glucose; and the change in uptake rate was similar for the four-cell system. This agrees with expectations, given the very low *K_m_* value for glucose uptake by *E. coli. K_m_* describes the concentration of a substrate at which uptake is half of *V*_max_; because *K_m_* is low relative to experimentally relevant glucose concentrations, most concentrations we calculated fell in the plateau region of the Michaelis–Menten curve where uptake rate is close to *V*_max_ and changes little with changing concentration (Figure 5).

## Discussion

Our results illustrate the degree to which cells in diffusion-limited environments such as microgravity can deplete growth substrates in their surrounding environment. The aim of this work was to generate quantitative information that would allow researchers to calculate the impact of these concentrations on cell behavior; and readers are welcome to implement the code we have developed (Supplementary material) to study similar processes in their organisms and media of interest.

Microgravity is not the only context in which these concentration gradients are relevant. An individual microbial cell growing in any fluid environment on Earth is inevitably surrounded by a diffusive boundary layer, and the smaller the organism, the larger the role this layer plays in determining chemical flux to the cell. Others have previously calculated the contributions of sinking, swimming, and turbulence for increasing nutrient fluxes to planktonic cells in environments with gravity, relative to diffusion alone (Purcell, 1978; Lazier and Mann, 1989; Karp-Boss et al., 1996; Guasto et al., 2012). In addition, substrate diffusion is a key process regulating growth, physical structure, and phenotypic heterogeneity in microbial colonies growing on solid substrates such as laboratory agar culture plates, and in the microbial communities of naturally occurring biofilms (Harcombe et al., 2014; Nadell et al., 2016; Julou et al., 2020).

The values we calculated for glucose concentrations surrounding a single cell using FDM differed substantially from those calculated using the analytical solution. This is likely because of the difference in the way the spatial dimensions are treated: as described above, the analytical solution assumes diffusion in all directions, whereas our implementation of FDM assumes diffusion only in the *x*–*y* plane, as though our single cell and its surroundings were a slice that is repeated indefinitely in the *z* direction. While in the single-cell case this scenario may overestimate the effect of glucose consumption on concentrations, in environments crowded with cells it becomes a closer approximation of reality. And environments with multiple cells in close proximity are precisely those that we aimed to investigate with this implementation.

It important to acknowledge that we defined the depletion zone arbitrarily as more than a 10% decrease from initial substrate concentration, but defining the biological effects of that particular concentration decrease is outside the scope of this study. We did examine the effect of concentration on uptake rate and found very little change, due to the low *K_m_* value for glucose by *E. coli*, 1.75 μM. This is notable especially given the fact that the FDM model likely overestimates the changes in glucose concentration. However, in scenarios different from those modeled here, such as other media and substrates, other cells, and other orientations, concentrations surrounding the cell might approach values that could influence uptake and/or growth rates. We used *E. coli* for this study because it is a well-studied model organism common in fundamental spaceflight research, but the influence of microgravity on bulk exchange is also of particular interest to microbial synthetic biology using diverse other chassis organisms (e.g., yeast, algae, *Streptomyces*, *Bacillus*). In the future such situations could be tested, as our model allows for parameters to be easily changed.

In the same vein, we have demonstrated the importance of interactions between neighboring cells. Even when cells are as far as 1,080 μm apart (from center to center of cell), the regions from which they consume glucose overlap enough to render their zones of depletion deeper (lower glucose concentrations) and wider (the radius of the 10% zone increases). The system of four cells told a similar story: groups of microbes located closely together have a synergistic effect that results in steeper concentration gradient than single cells standing alone in the medium. The difference we observed when increasing cell numbers from one to two to four, and from linear to square orientation, hinting at the results we might observe as a culture approaches high cell density and thousands of cells clustered with their zones overlapping, in the form of a three-dimensional colony. And the colony realm might in fact be very relevant to suspension culture in the space environment: when cells are non-motile and are not pushed away from each other by convection, cells in suspension culture in microgravity or in a RWV may spend most of their time not in isolation but rather as microcolonies.

Understanding the size and extent of the depletion zone has practical relevance to those seeking to understand the reasons for microbes to show altered phenotypes in microgravity, especially *via* experiments involving the simulation of microgravity on Earth. Rotating Wall Vessels (RWV) operate under the assumption that they provide a similar fluid environment to that of true microgravity by ensuring that cells travel in small circular paths that stay within their depletion zones (Hammond and Hammond, 2001; Klaus, 2001). Our results may be paired with calculations estimating the path traveled by microbial cells in RWVs (Allen et al., 2022) to aid in experimental design for future ground-based microgravity research.

Overall, our model provides important context for researchers attempting to understand what cells in suspension experience in microgravity and how these conditions affect them. Further research using the same methods to simulate other systems, possibly with an expansion to true 3D, would aid in improving our understanding of the big picture of the chemical environment surrounding a cell in microgravity. Our model could also be applied to an inverse problem, to calculate concentrations of waste products excreted by cells. Additionally, it could provide insight into communities that exchange growth nutrients, signaling compounds, or antibiotic toxins used in cell–cell competition—relationships that could be affected by these microgravity-induced concentration gradients. Pairing quantitative modeling tools with experimentation will allow us to probe more diverse questions and increase the value of spaceflight research.

## Data availability statement

The original contributions presented in the study are included in the article and/or Supplementary material; further inquiries can be directed to the corresponding author.

## Author contributions

JG and JL conceptualized the research question and wrote the manuscript. JG conceptualized and wrote the code for the project, and carried out the analysis and interpretation of results. JL provided the supervision. TL and JB gave advice on methodology and analysis, and reviewed and edited the manuscript. All authors contributed to the article and approved the submitted version.

## Funding

This research was funded by the NASA Biological and Physical Sciences Division (BPS) through the Space Life Sciences Training Program (SLSTP), the NASA Ames Office of STEM Engagement, the NASA BPS Space Biology Program through grant opportunity NNH20ZDA001N-SB, and a MathWorks Teaching Fellowship.

## Conflict of interest

The authors declare that the research was conducted in the absence of any commercial or financial relationships that could be construed as a potential conflict of interest.

## Publisher’s note

All claims expressed in this article are solely those of the authors and do not necessarily represent those of their affiliated organizations, or those of the publisher, the editors and the reviewers. Any product that may be evaluated in this article, or claim that may be made by its manufacturer, is not guaranteed or endorsed by the publisher.
