# Supplementary material for: The chemical neighborhood of cells in a diffusion-limited system
Source: Front Microbiol. 2023 Apr 18;14:1155726. doi: 10.3389/fmicb.2023.1155726 (PMC10151505; doi:10.3389/fmicb.2023.1155726)
Supplement: Supplementary file 1 [file Data_Sheet_1.docx]

%% clear

clc; close all; clear all;

%% chose orientation

%1= 4 cells spaced linearly

%2= 4 cells in box formation,

%3= two cells,

%4= 1 cell

cellchoice=4; %choose here

%% dimensions

X=2;

Y=2;

DZ=0.001; % 0.001 cm micron in the z direction for the slice

N=111;

DX=X/N;

DY=Y/N;

M=600000; % maximum number of iteration

DT =10; % seconds

%% positions for cells

spacing=1; %this times 2 is amt of DX between cells

center=56;

down=center-(spacing);

up=center+(spacing);

downdown=center-2*spacing;

upup=center+2*spacing;

%% initialize variables

InitialConcentrationmM=22; % initial concentration of nutrient in mM

Km=.00175; % Michaelis Menten constant in mM

cellvolL=(DX*DY*DZ)/1000; %cm3/(1000cm3/1L) = L

InitialConcentration=InitialConcentrationmM*cellvolL; %in mmoles

Km=Km*cellvolL;

allowchange=0.0001; %in mM

allowchange=allowchange*cellvolL;

Diff=6*10^(-6); % diffusivity in cm2/sec

alpha=Diff*DT; % cm2/sec time sec = cm2

%% choose Vmax value

value=1; %set to 1 for harcombe value; set 2 for other

if value==1

Vmax=10; % in mmol/g/hr

cellgrams=1*10^(-12); % e coli weighs one picogram

Vmax=Vmax*cellgrams; %in mmol/cell/hr by multipling by g/cell

Vmax=Vmax/(60*60); %in mmol/cell/sec

else

Vmax=197; % molecules/cell/sec

Vmax=Vmax/(6.02*10^(23)); % moles/cell/sec

Vmax=Vmax*1000; % in mmoles/cell/sec

end

%% initialize records

ncount=0;

loop=1;

countrecord=1;

a=1;

Vrec1=zeros(1,M);

Vrec2=zeros(1,M);

Vrec3=zeros(1,M);

Vrec4=zeros(1,M);

change=ones(1,M);

%% IC and BC

C(1:N,1:N) = InitialConcentration; %outer bounds will remain at this value

Crecord(:,:,1)=C;

%% finite difference

while loop==1

err=0;

Cold=C;

a=a+1;

for i = 2:N-1

for j = 2:N-1

delx=DX^2;

dely=DY^2;

scnddrvtvx=(Cold(i-1,j)-2*Cold(i,j)+Cold(i+1,j))/delx;

scnddrvtvy=(Cold(i,j-1)-2*Cold(i,j)+Cold(i,j+1))/dely;

C(i,j)=Cold(i,j)+(alpha*(scnddrvtvx+scnddrvtvy));

if cellchoice==1 %line

%%center down

cell1avg=(C(center-1,down)+C(center+1,down)+C(center,down-1)+C(center,down+1))/4;

C(center,down)=-Vmax*cell1avg/(cell1avg+Km);

%%center down down

cell2avg=(C(center-1,downdown)+C(center+1,downdown)+C(center,downdown-1)+C(center,downdown+1))/4;

C(center,downdown)=-Vmax*cell2avg/(cell2avg+Km);

%%center up

cell3avg=(C(center-1,up)+C(center+1,up)+C(center,up-1)+C(center,up+1))/4;

C(center,up)=-Vmax*(cell3avg)/(cell3avg+Km);

%%center up up

cell4avg=(C(center-1,center)+C(center+1,center)+C(center,center+1)+C(center,center-1))/4;

C(center,center)=-Vmax*(cell4avg)/(cell4avg+Km);

elseif cellchoice==2 %square

%%down down

cell1avg=(C(down-1,down)+C(down+1,down)+C(down,down-1)+C(down,down+1))/4;

C(down,down)=-Vmax*cell1avg/(cell1avg+Km);

%%up up

cell2avg=(C(up-1,up)+C(up+1,up)+C(up,up-1)+C(up,up+1))/4;

C(up,up)=-Vmax*(cell2avg)/(cell2avg+Km);

%%down up

cell3avg=(C(down-1,up)+C(down+1,up)+C(down,up-1)+C(down,up+1))/4;

C(down,up)=-Vmax*(cell3avg)/(cell3avg+Km);

%%updown

cell4avg=(C(up-1,down)+C(up+1,down)+C(up,down-1)+C(up,down+1))/4;

C(up,down)=-Vmax*cell4avg/((cell4avg)+Km);

elseif cellchoice==3

%center down

cell1avg=(C(center-1,down)+C(center+1,down)+C(center,down-1)+C(center,down+1))/4;

C(center,down)=-Vmax*(cell1avg)/(cell1avg+Km);

%center up

cell2avg=(C(center-1,up)+C(center+1,up)+C(center,up-1)+C(center,up+1))/4;

C(center,up)=-Vmax*(cell2avg)/(cell2avg+Km);

else

cell1avg=(C(center-1,center)+C(center+1,center)+C(center,center-1)+C(center,center+1))/4;

C(center,center)=-Vmax*(cell1avg)/(cell1avg+Km);

end

end

%% record uptake rate

if cellchoice==1

Vrec1=abs(Crecord(center,down,:));

Vrec2=abs(Crecord(center,downdown,:));

Vrec3=abs(Crecord(center,up,:));

Vrec4=abs(Crecord(center,center,:));

elseif cellchoice==2

Vrec1=abs(Crecord(down,down,:));

Vrec2=abs(Crecord(down,up,:));

Vrec3=abs(Crecord(up,up,:));

Vrec4=abs(Crecord(up,down,:));

elseif cellchoice==3

Vrec1=abs(Crecord(center,down,:));

Vrec2=abs(Crecord(center,up,:));

else

Vrec1=abs(Crecord(center,center,:));

end

%%record concentration

Crecord(:,:,a)=C;

end

if a>2

Crecordavg=Crecord;

if cellchoice==1

%%center down

cell1avg=(Crecord(center-1,down,:)+Crecord(center+1,down,:)+Crecord(center,down-1,:)+Crecord(center,down+1,:))/4;

Crecordavg(center,down,:)=cell1avg;

change=abs(Crecordavg(center,down,a)-Crecordavg(center,down,a-1));

elseif cellchoice==2

%%down down

cell1avg=(Crecord(down-1,down,:)+Crecord(down+1,down,:)+Crecord(down,down-1,:)+Crecord(down,down+1,:))/4;

Crecordavg(down,down,:)=cell1avg;

change=abs(Crecordavg(down,down,a)-Crecordavg(down,down,a-1));

elseif cellchoice==3

%center down

cell1avg=(Crecord(center-1,down,:)+Crecord(center+1,down,:)+Crecord(center,down-1,:)+Crecord(center,down+1,:))/4;

Crecordavg(center,down,:)=cell1avg;

change=abs(Crecordavg(center,down,a)-Crecordavg(center,down,a-1));

else

cell1avg=(Crecord(center-1,center,:)+Crecord(center+1,center,:)+Crecord(center,center-1,:)+Crecord(center,center+1,:))/4;

Crecordavg(center,center,:)=cell1avg;

change=abs(Crecordavg(center,center,a)-Crecordavg(center,center,a-1));

end

end

if(change>=allowchange) % allowed error limit is 1% of maximum concentration

ncount=ncount+1;

if(ncount>M) %if solution does not converge in M time steps

loop=0;

disp(['solution does not reach steady state in ',num2str(M),' time steps'])

end

else % if solution converges within M time steps

loop=0;

disp(['solution reaches steady state in ',num2str(ncount) ,'time steps'])

end

end

%% put back in mM from mmoles

C=C./cellvolL;

Crecord=Crecord./cellvolL;

%% average out cell location (Vmax)

Crecordavg=Crecord;

if cellchoice==1

%%center down

cell1avg=(Crecord(center-1,down,:)+Crecord(center+1,down,:)+Crecord(center,down-1,:)+Crecord(center,down+1,:))/4;

Crecordavg(center,down,:)=cell1avg;

%%center down down

cell2avg=(Crecord(center-1,downdown,:)+Crecord(center+1,downdown,:)+Crecord(center,downdown-1,:)+Crecord(center,downdown+1,:))/4;

Crecordavg(center,downdown,:)=cell2avg;

%%center up

cell3avg=(Crecord(center-1,up,:)+Crecord(center+1,up,:)+Crecord(center,up-1,:)+Crecord(center,up+1,:))/4;

Crecordavg(center,up,:)=cell3avg;

%%center up up

cell4avg=(Crecord(center-1,center,:)+Crecord(center+1,center,:)+Crecord(center,center+1,:)+Crecord(center,center-1,:))/4;

Crecordavg(center,center,:)=cell4avg;

elseif cellchoice==2

%%down down

cell1avg=(Crecord(down-1,down,:)+Crecord(down+1,down,:)+Crecord(down,down-1,:)+Crecord(down,down+1,:))/4;

Crecordavg(down,down,:)=cell1avg;

%%up up

cell2avg=(Crecord(up-1,up,:)+Crecord(up+1,up,:)+Crecord(up,up-1,:)+Crecord(up,up+1,:))/4;

Crecordavg(up,up,:)=cell2avg;

%%down up

cell3avg=(Crecord(down-1,up,:)+Crecord(down+1,up,:)+Crecord(down,up-1,:)+Crecord(down,up+1,:))/4;

Crecordavg(down,up,:)=cell3avg;

%%updown

cell4avg=(Crecord(up-1,down,:)+Crecord(up+1,down,:)+Crecord(up,down-1,:)+Crecord(up,down+1,:))/4;

Crecordavg(up,down,:)=cell4avg;

elseif cellchoice==3

%center down

cell1avg=(Crecord(center-1,down,:)+Crecord(center+1,down,:)+Crecord(center,down-1,:)+Crecord(center,down+1,:))/4;

Crecordavg(center,down,:)=cell1avg;

%center up

cell2avg=(Crecord(center-1,up,:)+Crecord(center+1,up,:)+Crecord(center,up-1,:)+Crecord(center,up+1,:))/4;

Crecordavg(center,up,:)=cell2avg;

else

cell1avg=(Crecord(center-1,center,:)+Crecord(center+1,center,:)+Crecord(center,center-1,:)+Crecord(center,center+1,:))/4;

Crecordavg(center,center,:)=cell1avg;

end

%% display figures

t=1:ncount;

t=t/DT;

%Vrec1=Vrec1*(6.02e+23)*cellvol_L;

%Vrec2=Vrec2*(6.02e+23)*cellvol_L;

%Vrec3=Vrec3*(6.02e+23)*cellvol_L;

%Vrec4=Vrec4*(6.02e+23)*cellvol_L;

X=X*10;

Y=Y*10;

if cellchoice==1

centerline=Crecordavg(center,:,a);

lineup=Crecordavg(:,up,a);

linedown=Crecordavg(:,down,a);

if a>M

c1(:)=Crecordavg(center,up,2:end);

c2(:)=Crecordavg(center,down,2:end);

c3(:)=Crecordavg(center,center,2:end);

c4(:)=Crecordavg(center,downdown,2:end);

else

c1(:)=Crecordavg(center,up,1:end-2);

c2(:)=Crecordavg(center,down,1:end-2);

c3(:)=Crecordavg(center,center,1:end-2);

c4(:)=Crecordavg(center,downdown,1:end-2);

end

f1= figure (1);

set(gcf,'color','white')

set(gca, 'color', [1 1 1])

plot(t,c1)

hold on

plot(t,c2)

plot(t,c3)

plot(t,c4)

xlabel('Time (s)')

ylabel('Concentration (mM)')

ylim([0 22])

title('Concentration over time, 0.2 mm from cell center') %%%%CHANGE VERYWHERE

hold off

f5=figure(5);

set(gcf,'color','white')

set(gca, 'color', [1 1 1])

plot (X, lineup)

xlabel('Space (\mm)','FontName','Times New Roman')

ylabel('Concentration (mM)','FontName','Times New Roman')

ylim([0 22])

title('lineup','FontName','Times New Roman')

f6=figure(6);

set(gcf,'color','white')

set(gca, 'color', [1 1 1])

plot (X, linedown)

xlabel('Y Space (mm)','FontName','Times New Roman')

ylabel('Concentration (mM)','FontName','Times New Roman')

ylim([0 22])

title('linedown','FontName','Times New Roman')

f7=figure (7);

set(gcf,'color','white')

set(gca, 'color', [1 1 1])

plot (1:N, centerline)

xlabel('Y Space (mm)','FontName','Times New Roman')

ylabel('Concentration (mM)','FontName','Times New Roman')

ylim([0 22])

title('centerline','FontName','Times New Roman')

elseif cellchoice==2

linedown=Crecordavg(down,:,a);

lineup=Crecordavg(up,:,a);

upline=Crecordavg(:,up,a);

downline=Crecordavg(:,down,a);

if (a>M)

c1(:)=Crecordavg(up,up,2:end);

c2(:)=Crecordavg(down,down,2:end);

c3(:)=Crecordavg(down,up,2:end);

c4(:)=Crecordavg(up,down,2:end);

else

c1(:)=Crecordavg(up,up,3:end);

c2(:)=Crecordavg(down,down,3:end);

c3(:)=Crecordavg(down,up,3:end);

c4(:)=Crecordavg(up,down,3:end);

end

f1= figure (1);

set(gcf,'color','white')

set(gca, 'color', [1 1 1])

plot(t,c1)

hold on

plot(t,c2)

plot(t,c3)

plot(t,c4)

xlabel('Time (s)')

ylabel('Concentration (mM)')

ylim([0 22])

title('Concentration over time, 0.2 mm from cell center')

hold off

f5=figure (4);

set(gcf,'color','white')

set(gca, 'color', [1 1 1])

plot (1:N, linedown)

xlabel('Y Space (mm)','FontName','Times New Roman')

ylabel('Concentration (mM)','FontName','Times New Roman')

ylim([0 22])

title('linedown','FontName','Times New Roman')

f5=figure (5);

set(gcf,'color','white')

set(gca, 'color', [1 1 1])

plot (1:N, lineup)

xlabel('Y Space (mm)','FontName','Times New Roman')

ylabel('Concentration (mM)','FontName','Times New Roman')

ylim([0 22])

title('lineup','FontName','Times New Roman')

f6=figure (6);

set(gcf,'color','white')

set(gca, 'color', [1 1 1])

plot (1:N, upline)

xlabel('Y Space (mm)','FontName','Times New Roman')

ylabel('Concentration (mM)','FontName','Times New Roman')

ylim([0 22])

title('upline','FontName','Times New Roman')

f7=figure (7);

set(gcf,'color','white')

set(gca, 'color', [1 1 1])

plot (1:N, downline)

xlabel('Y Space (mm)','FontName','Times New Roman')

ylabel('Concentration (mM)','FontName','Times New Roman')

ylim([0 22])

title('downline','FontName','Times New Roman')

elseif cellchoice==3

centerline=Crecordavg(center,:,a);

lineup=Crecordavg(:,up,a);

linedown=Crecordavg(:,down,a);

if (a>M)

c1(:)=Crecordavg(center,up,2:end);

c2(:)=Crecordavg(center,down,2:end);

else

c1(:)=Crecordavg(center,up,3:end);

c2(:)=Crecordavg(center,down,3:end);

end

f1= figure (1);

set(gcf,'color','white')

set(gca, 'color', [1 1 1])

plot(t,c1)

%hold on

%plot(t,c2)

xlabel('Time (s)')

ylabel('Concentration (mM)')

ylim([0 22])

title('Concentration over time, 0.2 mm from cell center')

%hold off

f5=figure(5);

set(gcf,'color','white')

set(gca, 'color', [1 1 1])

plot (1:N, lineup)

xlabel('Y Space (mm)','FontName','Times New Roman')

ylabel('Concentration (mM)','FontName','Times New Roman')

ylim([0 22])

title('lineup','FontName','Times New Roman')

f6=figure(6);

set(gcf,'color','white')

set(gca, 'color', [1 1 1])

plot (1:N, linedown)

xlabel('Y Space (mm)','FontName','Times New Roman')

ylabel('Concentration (mM)','FontName','Times New Roman')

ylim([0 22])

title('linedown','FontName','Times New Roman')

f7=figure (7);

set(gcf,'color','white')

set(gca, 'color', [1 1 1])

plot (1:N, centerline)

xlabel('Y Space (mm)','FontName','Times New Roman')

ylabel('Concentration (mM)','FontName','Times New Roman')

ylim([0 22])

title('centerline','FontName','Times New Roman')

else

centerline=Crecordavg(center,:,a);

lineup=Crecordavg(:,center,a);

if (a>M)

c1(:)=Crecordavg(center,center,2:end);

else

c1(:)=Crecordavg(center,center,3:end);

end

f1= figure (1);

set(gcf,'color','white')

set(gca, 'color', [1 1 1])

plot(t,c1)

%hold on

%plot(t,c2)

xlabel('Time (s)')

ylabel('Concentration (mM)')

title('Concentration over time, 0.2 mm from cell center')

%hold off

f5=figure(5);

set(gcf,'color','white')

set(gca, 'color', [1 1 1])

plot (1:N, lineup)

xlabel('Y Space (mm)','FontName','Times New Roman')

ylabel('Concentration (mM)','FontName','Times New Roman')

title('lineup','FontName','Times New Roman')

f7=figure (7);

set(gcf,'color','white')

set(gca, 'color', [1 1 1])

plot (1:N, centerline)

xlabel('Y Space (mm)','FontName','Times New Roman')

ylabel('Concentration (mM)','FontName','Times New Roman')

title('centerline','FontName','Times New Roman')

end

%% mich-ment plot

mmplotconc=linspace(0,22,1000);

mmplotuptake=Vmax.*(mmplotconc)./(Km+mmplotconc);

f2=figure(2);

set(gcf,'color','white')

set(gca, 'color', [1 1 1])

semilogx(mmplotconc,mmplotuptake)

xlabel('Log of Substrate Concentration (mM)','FontName','Times New Roman')

ylabel('Uptake Rate (mM/cell/sec)','FontName','Times New Roman')

title('Michaelis-Menten Curve','FontName','Times New Roman')

%% surfplot

f8=figure (8);

colormap hot

set(gcf,'color','white')

set(gca, 'color', [1 1 1])

surf(X,Y,Crecordavg(:,:,a))

clrbr=colorbar;

ylabel(clrbr,'Concentration (mM)','fontsize',12,'FontName','Times New Roman','Rotation',90);

hColourbar.Label.Position(1) = 3;

caxis([0 22])

xlabel('X (mm)','fontSize',12,'FontName','Times New Roman');

ylabel('Y (mm)','fontSize',12,'FontName','Times New Roman');

zlabel ('Concentration (mM)')

zlim([0 22])

title('Concentration of Glucose Across Space','fontsize',12,'FontName','Times New Roman');
