# Supplementary material for: The chemical neighborhood of cells in a diffusion-limited system
Source: Front Microbiol. 2023 Apr 18;14:1155726. doi: 10.3389/fmicb.2023.1155726 (PMC10151505; doi:10.3389/fmicb.2023.1155726)
Supplement: Supplementary file 3 [file Data_Sheet_3.pdf]

## Convergence Studies

We conucted convergence studies to ensure that the values we selected for spatial and temporal resolution did not affect the results of the Finite Difference Method calculations. While coarser resolution allows for faster computation, it might also lead to inaccurate results. We sought to find the levels of spatial and temporal resolution that were coarse enough to allow for feasible model run times (e.g. a few hours or less), while producing results that were similar to those produced by finer levels of resolution.

### *Time Step and Minimum Change*

Two variables that have the potential to affect our results are the length of the time step (*dt*) and the definition of steady state. We defined steady state as the timepoint at which the glucose concentration at the surface of the cell changed less than a given value per timestep; we called this value the "maximum acceptable change in concentration" or "Max Change." The value of $\delta t$ and the value of Max Change influence each other: for lower values of $\delta t$, changes in concentration at each time step are correspondingly lower, and the system reaches a given Max Change value more quickly.

In order to determine appropriate values for $\delta t$ and Max Change, we performed convergence studies. First, we began with four values of $\delta t$: 0.01 second, 0.1 second, 1 second, and 10 seconds. It is important to note that for smaller $\delta t$, computational time increases: iterations with a $\delta t = 0.01$ seconds took up to 10 hours to reach steady state. For the various $\delta t$ values, we tested the following Max Change values: 5 µM, 1 µM, 500 nM, 100 nM, 50 nM, and 10 nM. Figures 1-4 show how glucose conentrations at the cell surface (i.e., 1 spatial unit from the point source) changed with the changing values of $\delta t$ and Max Change. In all cases, we set $\delta x = \delta y = 0.05$ cm.

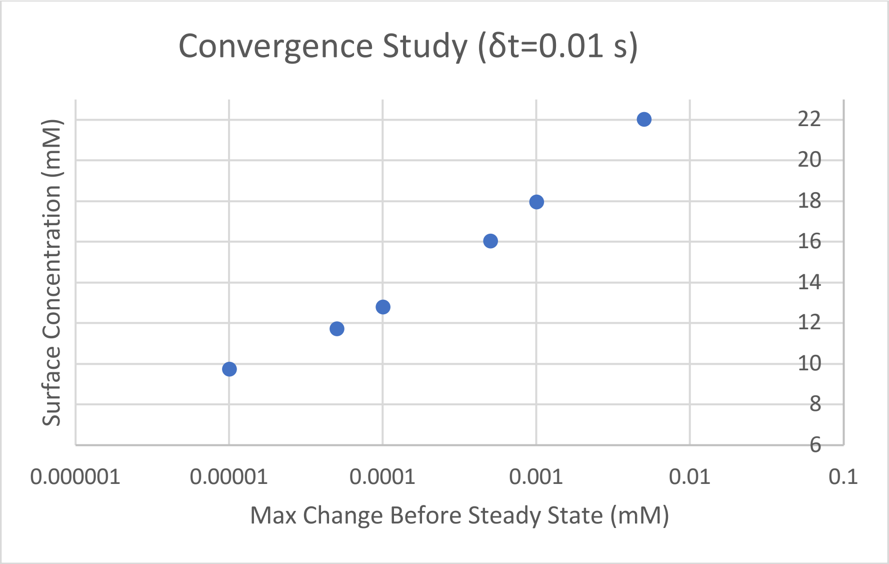


Figure 1. Concentration of glucose at the cell surface vs Max Change, for $\delta t = 0.01$ second. At lower values of Max Change, the simulation ran longer and reached a lower final glucose concentration.

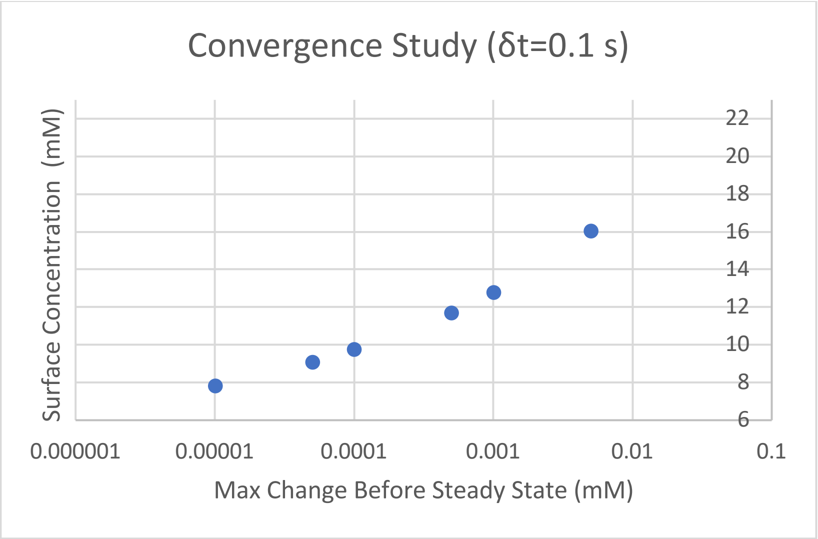


Figure 2. Concentration of glucose at the cell surface vs Max Change, for $\delta t = 0.1$ second.

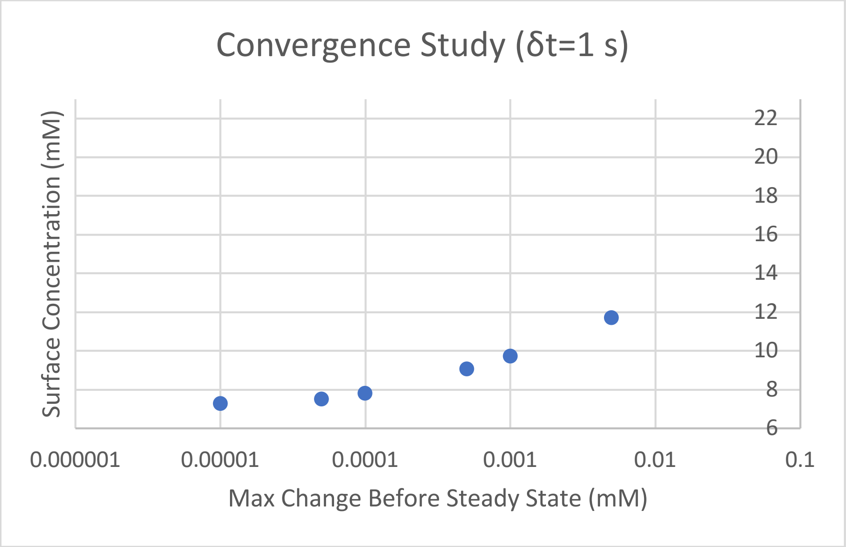


Figure 3. Concentration of glucose at the cell surface vs Max Change, for $\delta t = 1$ second.

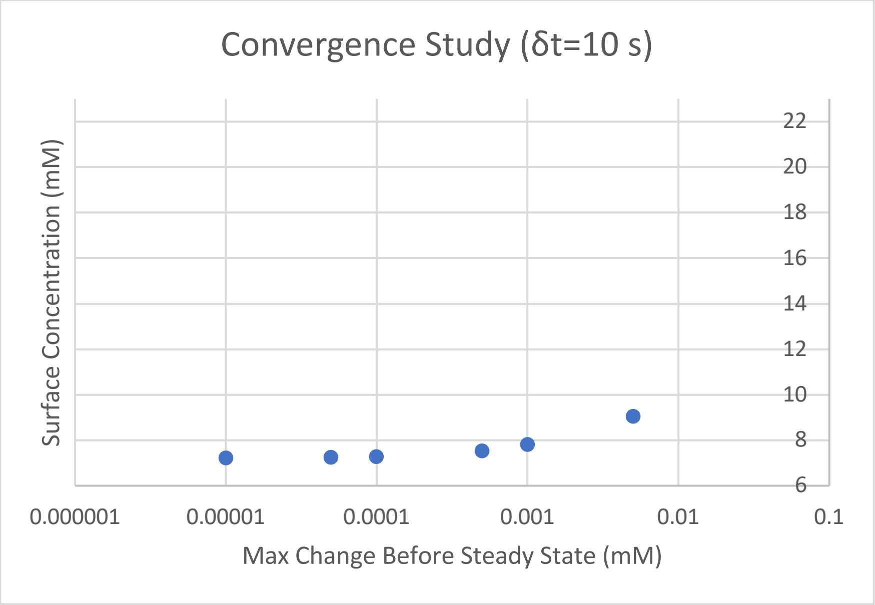


Figure 4. Concentration of glucose at the cell surface vs Max Change, for $\delta t$ = 10 seconds.

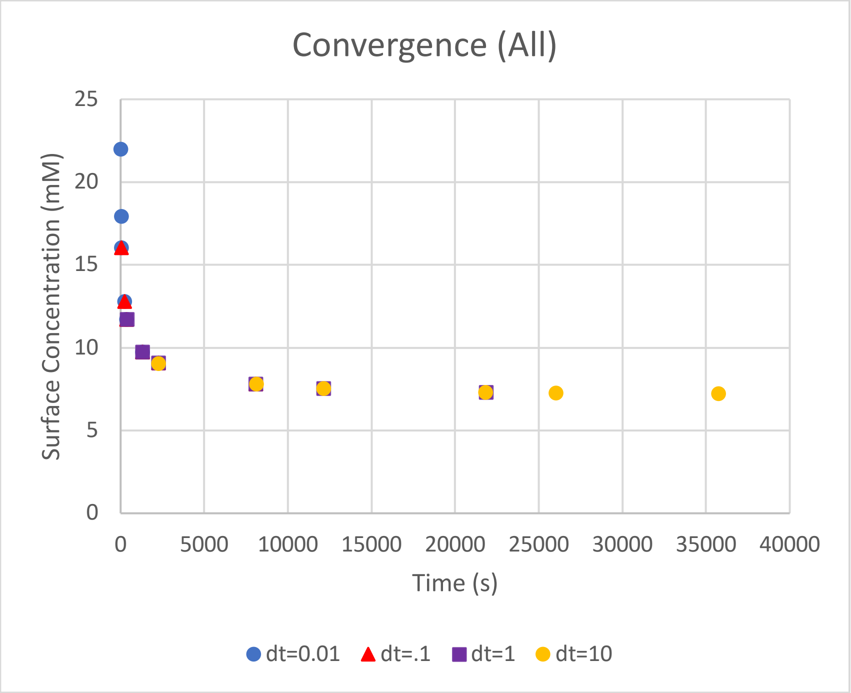


Figure 5. Concentration of glucose at the cell surface over simulated time (results from Figures 1-4). Color and shape represent the value of $\delta t$ used in the simulation.

Figure 5 shows the results from Figs. 1-4 in a single plot, with simulated time on the x-axis and final glucose concentration at the cell surface on the y-axis. Each point represents one run of the model with a particular Max Change and $\delta t$ value. The model was run until steady state was reached (according to the Max Change value). As might be expected, for some combinations of Max Change and $\delta t$, the

definition of steady state was achieved before the system was actually in steady state. Larger values of $\delta t$ allowed larger values of simulated time to be achieved (e.g. running the model for 3,500 iteraitons with $\delta t$ = 10 achieves 35,000 seconds in simulated time). However, all points fall along the same curve: it is likely that running the $\delta t$ = 0.01 model for long enough, and with a sufficient Max Change value, would achieve the same final cell surface glucose concentration as the $\delta t$ = 10 model.

Based on these results, we determined that we could comfortably use a time step of 10 seconds, as it allows for quicker processing in MATLAB, with the confidence that the results would converge at the same value. Additionally, we found using a Max Change value of 100 nM (0.0001 mM) is sufficient to reach steady state; smaller values (leading to longer model run times) produced nearly the same result.

### *System Size and Spatial Step*

Next, we performed convergence studies using these values of $\delta t$=10 seconds and Max Change=100 nM to determine optimal system size ($X$ and $Y$, the dimensions of the domain over which we solve) and the optimal spatial step size in the x and y directions ($\delta x$ and $\delta y$). We first tested a system size of $X$=$Y$=0.5 cm, 1 cm, 1.5 cm, 2 cm, 2.5 cm, and 3 cm while fixing $\delta x$ = 0.018 cm. We then tested a range of $\delta x$ values between 0.018 and 0.06 cm while fixing $X$=$Y$=1 cm. At all times, $\delta x$=$\delta y$.

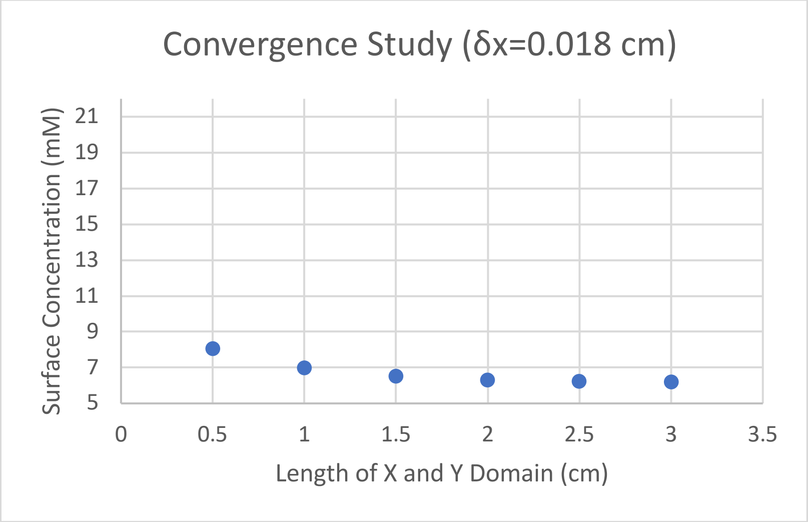


Figure 5. Concentration of glucose at the cell surface vs length of $X$ and $Y$ domain, with $\delta x = \delta y = 0.018$ cm.

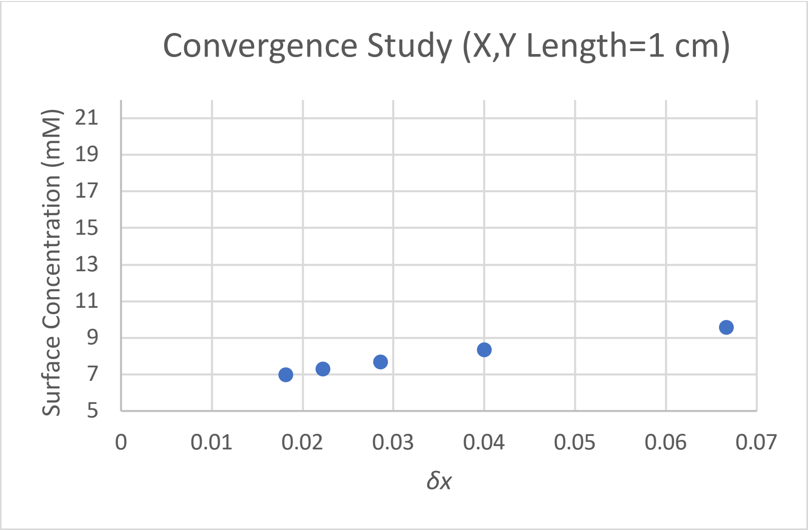


Figure 5. Concentration of glucose at the cell surface vs size of spatial step ($\delta x$), with $X = Y = 1$ cm.

As with the choice of $\delta t$, the choice of $\delta x$ and domain size had substantial effects on the time required to run the model. Based on the results shown above, we chose 2 cm for the domain size of $X$, $Y$, as the surface concentration calculated with $X$=2 cm converged within 5% of the previous value tested. The same is true for $\delta x = 0.018$ cm, which returned a value within 5% of the previous value tested. Therefore, we decided to perform all tests with a domain size of 2 cm by 2 cm, with $\delta x = \delta y = 0.018$ cm resulting in 111 steps each direction x and y.
